# Molecular Mechanisms Underlying Reciprocal Interactions Between Sleep Disorders and Parkinson’s Disease

**DOI:** 10.3389/fnins.2020.592989

**Published:** 2021-02-10

**Authors:** Zhengjie Yang, Xiaona Zhang, Chengqian Li, Song Chi, Anmu Xie

**Affiliations:** Department of Neurology, The Affiliated Hospital of Qingdao University, Qingdao, China

**Keywords:** Parkinson’s disease, glymphatic system, unfolded protein response, oxidative stress, sleep disruption

## Abstract

Sleep–wake disruptions are among the most prevalent and burdensome non-motor symptoms of Parkinson’s disease (PD). Clinical studies have demonstrated that these disturbances can precede the onset of typical motor symptoms by years, indicating that they may play a primary function in the pathogenesis of PD. Animal studies suggest that sleep facilitates the removal of metabolic wastes through the glymphatic system *via* convective flow from the periarterial space to the perivenous space, upregulates antioxidative defenses, and promotes the maintenance of neuronal protein homeostasis. Therefore, disruptions to the sleep–wake cycle have been associated with inefficient metabolic clearance and increased oxidative stress in the central nervous system (CNS). This leads to excessive accumulation of alpha-synuclein and the induction of neuronal loss, both of which have been proposed to be contributing factors to the pathogenesis and progression of PD. Additionally, recent studies have suggested that PD-related pathophysiological alterations during the prodromal phase disrupt sleep and circadian rhythms. Taken together, these findings indicate potential mechanistic interactions between sleep–wake disorders and PD progression as proposed in this review. Further research into the hypothetical mechanisms underlying these interactions would be valuable, as positive findings may provide promising insights into novel therapeutic interventions for PD.

## Introduction

Parkinson’s disease (PD) is one of the most prevalent neurodegenerative diseases worldwide and is associated with a significant burden on health care systems. Whereas the defining motor symptoms and pathological features of the disease, such as the loss of dopaminergic neurons and Lewy pathology, are attributed to the accumulation of alpha-synuclein, PD is recognized as a multisystem disorder associated with diverse non-motor symptoms (Munhoz et al., 2015). Disturbances of sleep patterns are among the most burdensome defects and manifest as insomnia, parasomnia, disturbances of wakefulness, or disruption of circadian rhythms, occurring with high prevalence during the course of the disease (Politis et al., 2010; Chahine et al., 2017). Subjective sleep complaints have been found in nearly half of newly diagnosed PD patients and result in prominent adverse effects on their quality of life (Breen et al., 2014). In a multicenter survey assessing the incidence of non-motor complaints in PD patients, sleep problems were present in 64.1% of subjects, making it the second most frequent non-motor symptom (Barone et al., 2009). Other studies have reported the prevalence of sleep disruptions in PD patients to be even higher, up to 73.7% (Maeda et al., 2017). Interestingly, accumulating evidence suggests that the onset of sleep–wake disturbances occurs years before the typical motor manifestations of PD, providing promising insight into the potential disease-modifying effect of sleep disturbances (Hastings and Goedert, 2013; Videnovic et al., 2014; Abbott and Videnovic, 2016). The rapid eye movement (REM) sleep behavior disorder (RBD), characterized by dream enactment behavior and loss of atonia during REM sleep, has been widely investigated over the past decades as a prodromal syndrome for PD which may antecede motor symptoms for years (Reichmann, 2017). Recently, a systematic review of 51 longitudinal studies indicated that an ongoing diagnosis of RBD is associated with a progressively increasing cumulative risk of neurodegenerative disorders (5-year risk: 33.5%; 10.5-year risk: 82.4%; and 14-year risk: 96.6%), with the majority of participants studied developing PD (43.6%) and Lewy body dementia (25.0%) (Galbiati et al., 2019). Recent studies suggest the occurrence of other subtype of sleep disorders may also precedes PD for years. For example, a population-based longitudinal study of 91,273 participants with non-apnea sleep–wake disorders but without PD revealed that sleep disorders might be an independent contributor associated with higher risk of PD, according to analysis by a Cox regression model with multivariate adjustment (Hsiao et al., 2017). Further subtype analysis by the authors revealed that chronic insomnia conferred the highest risk of PD. A population-based study over 64,855 person-years demonstrated 75 incident parkinsonism cases, 62.7% of whom exhibited PD during the 13 years of follow-up (Lysen et al., 2019). Statistical analysis indicated that worse sleep quality and shorter sleep duration were associated with a higher risk of incident parkinsonism within the first 2 years.

Findings from post-mortem and animal experiments corroborate this hypothesis. Elderly individuals without PD suffering from years of sleep fragmentation were found to have a greater risk of PD-related pathology and dopaminergic neuron loss after death (Sohail et al., 2017). Experimentally induced disturbances of sleep patterns have also been shown to impact the efficiency of the metabolic clearance system (Xie et al., 2013), resulting in disruption of protein homeostasis and increased oxidative stress in the central nervous system (CNS) (Naidoo et al., 2008; Singh et al., 2008; Andreazza et al., 2010; Lima et al., 2014; Zhang et al., 2014; Kröller-Schön et al., 2018; Rodrigues et al., 2018); all of these factors have been proposed to contribute to the progression of PD. Furthermore, the disruption of circadian rhythm has also been suggested to relate to neurodegeneration in animal models (Mattis and Sehgal, 2016).

**FIGURE 1:**
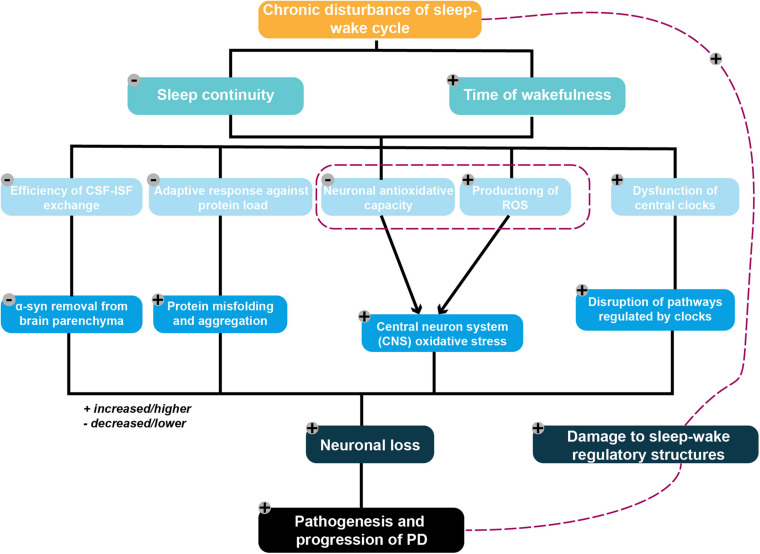
Framework of the potential mechanistic interplay between sleep–wake disruption and Parkinson’s disease (PD) pathogenesis. Chronic disruption of the sleep–wake cycle characterized by an increased waking time and decreased sleep continuity are proposed to serve as contributing factors to neuronal loss. These ultimately result in PD pathogenesis *via* several potential mechanisms, including the following: (1) diminishing the removal of metabolic wastes from the brain by adversely affecting the efficiency of the glymphatic system, resulting in excessive accumulation of the PD-promoting protein alpha-synuclein; (2) placing an excessive burden on the protein folding and degradation system, which is already overloaded in elderly individuals, resulting in the acceleration of protein aggregation and a positive feedback loop; (3) reducing neuronal antioxidative capacity, with the resulting increased ROS production triggered by excessive neuronal energy consumption subsequently leading to increased oxidative stress in the CNS; and (4) aggravating the disruption of pathways regulating the central circadian clock following dysfunction of these clocks. PD-related pathophysiological processes may also damage sleep–wake regulatory circuits, thereby exacerbating any existing disruptions to the sleep–wake cycle.

As such, an increasing number of studies suggest that PD-related pathology *per se* can contribute to the disturbance of sleep and of circadian rhythms. Increased deposition of alpha-synuclein related to PD was found in structures responsible for the regulation of sleep patterns, in line with disruptions to sleep and circadian rhythm (Rothman and Mattson, 2012; Kalaitzakis et al., 2013). Taken together, existing findings broaden our perspective on the potential mechanisms that may underlie reciprocal interactions between PD pathogenesis and sleep–wake disruptions. The purpose of this review, therefore, is to provide a framework and a comprehensive overview of the candidate molecular mechanisms proposed to underlie such a bidirectional relationship (Figure 1). In particular, it focuses on the potential molecular mechanism by which sleep disturbances lead to the pathogenesis and development of PD.

## Sleep–Wake Disruption Is a Contributing Factor to the Pathogenesis and Progression of Parkinson’s Disease

### Accumulation of Abnormal Proteins: The Endoplasmic Reticulum Stress Response and the Glymphatic System

#### Er Stress Response

Protein misfolding, accumulation, and aggregation play a fundamental role in the pathogenesis of neurodegenerative diseases, including Alzheimer’s disease (AD) and PD. Intracellular protein homeostasis, or proteostasis, is maintained by an integrated cellular quality control system based on the proper functioning of the endoplasmic reticulum (ER), which protects cells against stress induced by protein aggregation (Kaushik and Cuervo, 2015). ER stress is triggered by the aggregation of misfolded proteins and leads to activation of the unfolded protein response (UPR; also called the ER stress response) (Schröder and Kaufman, 2005a, b), an adaptive, coordinated response initiated by three transmembrane proteins. The UPR attenuates protein load *via* several different pathways, including diminishing protein translation *via* phosphorylation of eukaryotic initiation factor 2α (eIF2α) and enhancing protein modification and maturation *via* upregulation of the expression of specific genes, in coordination with elevated expression of ER chaperones (Szegezdi et al., 2006).

Given that one important protective function of the UPR is to maintain ER protein hemostasis by attenuating protein load, comprising a homeostatic response to counteract ER stress, it is unsurprising to note that dysfunction of the UPR can therefore give rise to disease (Scheper and Hoozemans, 2015). In neurodegenerative disease, disturbed activation of the UPR is correlated with ER dysfunction, which leads to synaptic dysfunction and neuronal malfunction. *In vitro* models have provided the majority of the evidence implicating UPR mediators in PD. Analysis of cortical neurons derived from induced pluripotent stem (iPS) cells from PD patients haboring alpha-synuclein (α-syn) mutations were found to show an accumulation of ER-associated degradation (ERAD) substrates, indicating impairment of the UPR and induction of ER stress (Chung et al., 2013). Consistent with these findings, a post-mortem study demonstrated the presence of UPR mediators in substantia nigra obtained from PD patients (Hoozemans et al., 2007). Furthermore, an immunohistochemical study found increased phosphorylation of pancreatic ER kinase (PKR)-like ER kinase (PERK) and eIF2α in dopaminergic neurons in the substantia nigra (SN) of PD patients compared with age-matched controls, and notably, increased α-syn immunoreactivity was shown to be colocalized with these UPR mediators. Taken together, these studies indicate that chronic ER stress caused by the accumulation of misfolded proteins and by disturbance of the UPR may comprise part of the underlying mechanism of PD, as well as contributing to abnormal aggregation of the key PD-related protein α-syn.

Acute sleep deprivation has been shown to cause activation of the ER stress response in the cerebral cortex of young mice (Naidoo et al., 2005). In contrast, aged mice were found to demonstrate significantly suppressed UPR activation in response to sleep disturbance (Naidoo et al., 2008). Whereas sleep deprivation did not increase expression of the major ER chaperone Ig binding protein (BiP) in aged mice, levels of BiP in young mice increased after 6 h of acute sleep deprivation. Additionally, the authors of the study found that levels of pro-apoptotic proteins increased in the cerebral cortex of aged mice, suggesting that the ER stress response is attenuated by aging (Naidoo et al., 2008). Similar to aged mice, patients with PD consistently show well-documented evidence of fragmented sleep (Chahine et al., 2017; Pillai and Leverenz, 2017). Therefore, it is reasonable to hypothesize that sleep disturbances characterized by sleep loss and fragmentation place an excessive burden on the proteostasis system, which is already overloaded in elderly patients. This results in the acceleration of protein aggregation, thereby forming a positive feedback loop. Interestingly, a recent study of BiP heterozygous mice reported a deterioration of the age-related sleep–wake phenotype, which was especially characterized by a fragmented sleep–wake cycle associated with decreased expression of BiP (Naidoo et al., 2018). These results indicate that impairment of the UPR may in turn contribute to damage to sleep architectures. Taken together, these findings suggest that the interaction between sleep disruption and maladaptive proteostatic regulation contributes to the development and progression of PD, but further efforts are needed to clarify how these three aspects interrelate.

Furthermore, increasing evidence has demonstrated that exposure to intermittent cyclical hypoxia/reoxygenation, which models sleep apnea-induced ER stress in specific brainstem motor neurons in adult mice as well as hippocampus and prefrontal cortex in young mice (Zhu et al., 2008; Cai et al., 2014), is ultimately implicated in impaired motor and cognitive function (Cai et al., 2013). This suggests that sleep-related breathing disorders, which are another sleep-disorder phenotype alongside sleep loss and fragmentation, are an aging-independent mechanism for inducing ER stress-mediated neuronal impairment. However, further studies are needed to determine whether the impairment observed in the mammalian models is also evident in patients with sleep apnea.

Evidence from human studies lending further support to the these hypotheses has started to emerge. Recently, a study conducted on 246 elderly patients without PD revealed that greater sleep fragmentation was associated with a higher risk of Lewy pathology and dopaminergic neuron loss (Sohail et al., 2017). Analyses with logistic regression models suggested that each 1 standard deviation greater sleep fragmentation led to a >40% higher likelihood of PD-related pathologic alterations. However, longitudinal studies that use non-invasive imaging techniques to evaluate correlations between sleep disorders and PD pathology still need to determine the directionality of the causal relationship between these two conditions. Additionally, to corroborate the hypotheses presented, assessments of cerebrospinal fluid (CSF) α-syn level in sleep disorder patients without PD are required to quantify α-syn deposition.

#### Glymphatic System

The glymphatic system is a newly described brain-wide network of perivascular spaces linked by astrocytic aquaporin-4 (AQP-4) water channels that facilitates the clearance of interstitial soluble protein and metabolic wastes from the brain. Cerebrospinal fluid enters the parenchyma *via* perivascular spaces that surround the penetrating arteries, while brain interstitial fluid exits through perivenous spaces (Iliff et al., 2012). Evidence suggests that impairment of this system is associated with the formation of neurotoxic protein aggregates that are implicated in the progression of neurodegenerative disease (Kress et al., 2014; Verheggen et al., 2018).

Sleeping mice were reported in one study to demonstrate an increased glymphatic clearance efficiency compared with freely behaving awake mice, with enhanced removal of the potentially neurotoxic metabolic products that typically accumulate during wakefulness (Xie et al., 2013). Based on real-time assessment of tetramethylammonium diffusion and two-photon imaging in live mice, the authors demonstrated that the brains of sleeping mice showed a 60% increase in the volume of the interstitial space compared with that of awake mice, resulting in increased convective flow between the CSF and interstitial fluid (ISF). The removal of potentially neurotoxic waste products increased sharply during sleep, as evidenced by a twofold faster removal of Aβ compared with awake mice, consistent with an increased efficiency of CSF–ISF exchange. Interestingly, other sleep-like states have also been found to exhibit an increased clearance efficiency. For example, studies have confirmed that glymphatic transport is enhanced during anesthesia, in line with results from animal studies (Benveniste et al., 2017; Ratner et al., 2017). Given these observations, sleep-like brain activity rather than sleep itself is assumed to determine the volume of the interstitial space, further impacting the efficiency of glymphatic solute clearance (Sundaram et al., 2019). This conclusion implies that factors correlated with the alteration of sleep architecture, including RBD and circadian rhythm, would be expected to subsequently induce or diminish the glymphatic disturbance.

While the clearance of α-syn *via* the glymphatic system has not yet been investigated, Hoshi and colleagues showed a negative correlation between AQP-4 expression and α-syn deposition, suggesting that a defined population of AQP-4 proteins serves as mediators *via* which the glymphatic system modifies α-syn deposition in the neocortex of patients with PD (Hoshi et al., 2017). A recent study indicated that glymphatic system dysfunction may be implicated in accelerating brain α-syn aggregation and the progression of α-syn-related pathology (Zou et al., 2019). Ligation of the deep cervical lymph nodes (LDclns) blocks meningeal lymphatic drainage in both wild-type and A53T-ligated mice and is associated with almost complete blockage of the glymphatic influx of a CSF tracer (Zou et al., 2019). The intensities of both α-syn-positive intercellular inclusions in neurons and extracellular α-syn aggregations in the perivascular space were significantly increased in LDclns mice compared with controls (Zou et al., 2019). These results indicate that an inefficiency of glymphatic solute clearance is implicated in the excessive accumulation of α-syn and that such clearance deteriorates after ligation of the lymph nodes. As a cautionary note, the LDclns mice in this study showed an increased number and distribution of gliocytes, which were associated with a fiercer neuroinflammatory response. This adds further support to the existing theory that excessive α-syn aggregation induces reactive gliosis, triggering the production of inflammatory cytokines, resulting in aggravated neuronal degeneration and apoptosis (Kempuraj et al., 2017; Wong and Krainc, 2017). Previous studies have confirmed that reactive astrogliosis leads to an impairment of AQP-4 polarization, subsequently causing glymphatic clearance inefficiency (Iliff and Nedergaard, 2013; Verkhratsky et al., 2015). Based on these findings, we hypothesize a vicious spiral in which dysfunction of glymphatic clearance is a crucial factor in the exacerbation of α-syn accumulation in the brain parenchyma. This accumulation leads to increased levels of reactive astrogliosis and AQP-4 depolarization, aggravating glymphatic system deficits and promoting PD-related pathology as a result.

Dopamine (DA) and noradrenaline receptors appear to drive the brain state-dependent alterations of the interstitial space volume, ultimately modulating CSF–ISF exchange (Benveniste et al., 2017). During waking states, levels of DA and adrenaline increase (Murillo-Rodríguez et al., 2009; España et al., 2016), amplifying both DA and catecholamine volume transmission signaling (Fuxe et al., 2015). DA volume transmission has been shown to be strongly involved in neuron–glia interactions (Fuxe et al., 2015), while suppression of adrenergic signaling in the brain during the waking state results in augmented glymphatic tracer influx (Xie et al., 2013). Therefore, it is reasonable to hypothesize that sleep disorders characterized by an extended waking duration and increased sleep fragmentation may show reduced α-syn removal *via* the glymphatic clearance system due to enhanced DA and catecholamine volume transmission during waking states.

### Promotion of Oxidative Stress

The accumulation of reactive oxygen species (ROS) following cellular redox imbalance leads to mitochondrial dysfunction and oxidative stress in the brain. These processes are also thought to play important roles in the pathogenesis of PD, independent of the effects of aggregation and deposition of misfolded proteins in the CNS (Phillipson, 2017). Data collected from preclinical PD patients indicates that they exhibit an elevated level of cellular oxidative stress, which occurs prior to significant neuronal loss (Trist et al., 2019). Mounting evidence from both *in vitro* and *in vivo* studies indicates that redox imbalance is a contributor to the cascade underlying dopaminergic neurodegeneration; moreover, strategies to restore redox homeostasis reverse this neurodegenerative process (Devos et al., 2014; Filograna et al., 2016; Biosa et al., 2018). Excessive accumulation of ROS is thought to adversely affect the chemical modification and oxidation of DNA, lipids, and proteins at the molecular level and contribute to apoptosis, cytoplasmic cell death, and autophagic cell death at the cellular level (Umeno et al., 2017; Morris et al., 2018). Damage to mitochondria, especially inhibition of the electron transport chain leading to chronic production of ROS, has also been observed in the SN of PD patients (Schapira et al., 1990). In addition, electron transport chain inefficiency caused by chronic administration of the complex I inhibitor rotenone was shown to be an important contributing factor to the aggregation of proteinaceous inclusions resembling Lewy bodies, as well as to degeneration of the SN in animal models (Cannon et al., 2009).

Reactive oxygen species and reactive nitrogen species (RNS) are unavoidable byproducts of a variety of essential physiological reactions in all aerobic organisms. An antioxidant system composed of antioxidants and protective enzymes is responsible for providing the organism with an antioxidant defense, which involves scavenging free radicals and transforming them into more stable chemical forms. Once the excessive release of reactive species surpasses the protective capacity of the antioxidant system, oxidative stress occurs. This potentially leads to adverse effects on pivotal cellular constituents, including lipids, proteins, and nucleic acids, affecting both the structure and function of neurons, and finally initiating cell death and hence causing neurodegeneration (Sultana and Butterfield, 2010; Jiang et al., 2016; Trist et al., 2019).

Sleep is thought to promote the removal of excessive reactive species accumulated during the waking state by decreasing oxidant production and upregulating the efficiency of antioxidant mechanisms (Reimund, 1994). Sleep deprivation has been implicated in increased cerebral oxidative stress in a multitude of animal studies (Singh et al., 2008; Andreazza et al., 2010; Lima et al., 2014; Zhang et al., 2014; Kröller-Schön et al., 2018; Rodrigues et al., 2018), indicating a potential mechanistic correlation between sleep deprivation and the pathogenesis of PD. Sleep deprivation for 8 h daily over 3 days in mice was found to increase the extent of oxidative stress and the acetylation of mitochondrial proteins in locus coeruleus neurons, in line with an elevated concentration of superoxide (Zhang et al., 2014). Another study found that mice experiencing either 48 or 72 h paradoxical sleep deprivation (PSD) demonstrated increased lipid peroxidation in striatum neurons, indicating an elevated level of oxidative stress in this brain region. It should be noted that nitrite level was selectively attenuated in the striatum in the 72-h PSD group, while it was reduced in hippocampus and prefrontal cortex in both the 72- and 48-h PSD groups (Lima et al., 2014). The unaltered level of nitrite in the striatum in the 48-h group indicates that this structure is less susceptible to oxidative stress and is only affected by prolonged sleep deprivation. A meta-analysis of data extracted from 44 studies using either total sleep deprivation (TSD) or PSD protocols suggested that the brain regions with elevated rates of reactive species production during periods of extended wakefulness are those which primarily show signs of oxidative stress, namely the hypothalamus and hippocampus (Villafuerte et al., 2015). The results of this study further suggest that REM sleep plays a significant role in the antioxidant function of sleep, while that of slow wave sleep is less important given the discrepant results obtained from the TSD experiments.

Several hypotheses have emerged to explain the potential mechanisms underlying sleep loss and oxidative stress in the CNS. One such hypothesis focuses on the reduced expression of genes that occurs during adaptive response to stress. In line with this theory, 7 days of sleep deprivation was found to attenuate the expression of transcriptor Nrf-2 (Xue et al., 2019), a transcription factor modulating basal and inducible expression of several antioxidant and detoxification enzymes, such as superoxide dismutase and catalase (Rodrigues et al., 2018). Sleep deprivation has also been reported to result in an inefficient response of cellular scavenger mechanisms to reactive species, accompanied by a reduction in the activity of mitochondrial membrane binding protein sirtuin type 3 to upregulate antioxidant defenses (Zhang et al., 2014). Further studies are warranted to clarify the molecular mechanisms underlying the correlation between sleep deprivation and increased oxidative stress in the brain.

### Circadian Rhythm Dysfunction

Studies conducted on night-shift workers and delayed sleep phase disorder patients have found that they exhibit altered melatonin profiles, as well as suppressed nocturnal melatonin secretion (Gibbs et al., 2002; Borugian et al., 2005; Marie Hansen et al., 2006; Micic et al., 2015). This reduction of melatonin production may be attributable to reduced rhythmicity of the central circadian pacemaker in the hypothalamic suprachiasmatic nucleus (SCN), which regulates melatonin secretion driven by the light–dark cycle (Phillipson, 2017). In line with this theory, mice treated with continuous light exposure for 24 weeks demonstrated a significant reduction of the rhythm and amplitude of SCN activity, which rapidly recovered upon return to a standard light–dark cycle (Lucassen et al., 2016). Melatonin has been shown to be multifunctional molecule that is pivotal in modulating the antioxidative system of cells, especially by acting as a powerful free-radical scavenger (Reiter et al., 2010). *In vitro* studies have also revealed that melatonin inhibiting the assembly of α-syn fibrils and destabilizing already formed fibrils, thereby preventing α-syn-induced cytotoxic events leading to neuronal death (Ono et al., 2012). Independent of its direct inhibitory and destabilizatory effects on fibril formation, studies have demonstrated that melatonin participates in multiple mechanisms implicated in neuroprotective processes, such as in the vitagene system, which includes sirtuins, heat shock proteins and heme oxygenase-1 (Gallardo-Fernandez et al., 2019). The vitagene system has been proposed to exert a pronounced neuroprotective effect given its positive role in attenuating α-syn misfolding and oxidative stress that would otherwise adversely affect neurons (Wang et al., 2007; Ebrahimi-Fakhari et al., 2012). *In vitro* investigations have demonstrated that melatonin significantly increases expression of sirtuin type 2 and nuclear transcription of Nrf-2, adding further support to the idea that melatonin acts as neuroprotective molecule on multiple levels (Gallardo-Fernandez et al., 2019; Yu et al., 2019). In conclusion, long-term delayed sleep phase or shift work has been shown to suppress melatonin secretion, thereby creating a vulnerability to alpha-synucleinopathy.

Mounting evidence suggests that malfunctioning endogenous clocks are a direct contributing factor to the pathogenesis and progression of neurodegenerative disease through their adverse effects on gene transcription and antioxidative processes. Myriad animal and human studies have reported that mistimed sleep leads to significantly reduced expression of circadian transcripts in the brain (Archer and Oster, 2015). The altered expression of clock genes that are fundamental to maintaining circadian rhythm has been recognized as playing a causal role in the induction of neurodegeneration (Hood and Amir, 2017). For example, one studying using genetic analysis identified the single-nucleotide polymorphisms in the *ARNTL* and *PER1* genes as potential contributors to an increased risk of PD (Gu et al., 2015). Endogenous clock dysfunction in mutant drosophila has been correlated with the loss of dopaminergic neurons and results in early development of age-related locomotor deficits *via* the activation of proapoptosis pathways and increased oxidative stress (Vaccaro et al., 2017). A multitude of findings add further support to the idea that the suppressed expression of clock genes contributes to accelerated neuronal degeneration, driven by weakened antioxidant defenses and the resulting increased oxidative stress in neurons. Mice with a brain and muscle Arnt-like protein 1 (BMAL1) deletion demonstrated significantly elevated rates of cellular oxidative damage, consistent with reduced expression of Nrf-2, the major antioxidative regulatory factor (Lee et al., 2013). Further chromatin immunoprecipitation analysis in this study revealed that BMAL1 directly regulates the expression of Nrf-2 *via* a transcriptional mechanism. Supplementation of BMAL1-deficient mice with the antioxidant *N*-acetyl-L-cysteine significantly reversed their decreased longevity and age-related pathologies, supporting a casual link between clock genes and an increased oxidative stress response, which eventually leads to aging and age-related diseases (Kondratov et al., 2009). A recent study linked suppressed BMAL1 function to a reduced level of silent information regulator, an aldehyde dehydrogenase-dependent deacetylase that deacetylates BMAL1 and PER2 proteins, thereby modulating the expression of clock genes and affecting circadian rhythms (Wang et al., 2018). Rats treated with 6-hydroxydopamine (6-OHDA) demonstrated significant circadian misalignment, augmented ROS accumulation, and attenuated expression of clock-controlled antioxidant genes. An *in vitro* study of 6-OHDA-treated SH-SY5Y cells reported an elevated level of acetylated BMAL1 and a decreased level of SIRT1 protein, suggesting that the molecular mechanism underlying the circadian dysfunction-induced impairment of antioxidative defenses in brain neurons is closely associated with the SIRT1–BMAL1 pathway (Wang et al., 2018). Studies on transgenic mice also indicate that the circadian disruption contributes to PD pathogenesis *via* exaggerating 1-methyl-4-phenyl-1,2,3,6-tetrahydropyridinen (MPTP)-induced neurotoxicity. Mice that underwent circadian disruption for 60 days before receiving MPTP treatment exhibited a worsening of their locomotor and cognitive deficits compared with those kept on a regular sleep-wake cycle, secondary to a significant reduced number of tyrosine hydroxylase (TH)-positive dopaminergic neurons and intense glial response (Lauretti et al., 2017). Another study demonstrated that inactivation of BMAL1 in the MPTP-treated mice resulted in significant motor deficit, further *in vivo* and *in vitro* study showed a significant reduction of dopaminergic neurons in the substantia nigra pars compacta, decreased level of the TH protein and the dopamine content in the striatum, and increased activation of glial-mediated neuroinflammation (Liu et al., 2020). Taken together, these findings suggest that dysfunction of the molecular clock attenuates the expression of redox defense genes, weakening the antioxidative response and thereby increasing the accumulation of reactive species. Meanwhile, the activation of gial cells and expression level of inflammatory factors will increase responsing to the circadian disruption. These in turn create neurotoxic conditions that may result in mitochondrial damage and microglial overactivation, leading to the loss of dopaminergic neurons *via* activation of proapoptotic or pro-inflammatory pathways, and ultimately accelerating the pathogenesis and progression of neurodegenerative disease.

Although these studies have provided novel insights into the mechanisms underlying the correlation between circadian disturbance and neurodegenerative disease, there are still several gaps remaining in our knowledge that warrant further investigation. Given that the glymphatic system promotes the effective clearance of accumulated neurotoxic metabolic products from the brain and generally operates during sleep, it is of major importance to clarify whether clock gene deletion impairs the efficiency of such glymphatic clearance. Furthermore, it is important to ascertain to what extent glymphatic clearance efficiency is impacted by disturbances to the circadian system. One proposal is that disturbances to the 24-h circadian cycle involving prolonged nighttime waking triggered by decreased clock gene expression could give rise to malfunction of the glymphatic system, thereby promoting the accumulation of PD-promoting metabolites in the brain parenchyma and speeding disease progression (Sundaram et al., 2019). Additionally, investigations of circadian α-syn dynamics in the CSF and ISF of the human brain are hampered by the lack of accurate measurements of glymphatic operation. Although a multitude of animal studies have shed light on the role of sleep as a regulator of metabolic waste clearance from the brain, further studies should transfer these investigations to human models to explore more reliable and valid methods of measuring glymphatic efficiency (Sundaram et al., 2019).

## Sleep and Circadian Rhythm Disturbance Resulting From PD-Related Processes

Mounting lines of evidence have revealed a potential causal link between PD-related processes and circadian disturbance in PD patients. Epidemiological studies conducted on early, untreated PD patients and age-matched controls have demonstrated a tendency for increased sleep latency, decreased sleep efficiency, and reduced REM duration in PD patients (Bušková et al., 2011; Yong et al., 2011; Breen et al., 2014). These results are in line with those of transgenic rodent models overexpressing α-synuclein, which exhibit increased non-REM sleep, decreased REM sleep, and disturbed oscillatory electroencephalographic activity reminiscent of PD (McDowell et al., 2014). Additionally, changes to sleep architecture have been shown to worsen as PD progresses (Diederich et al., 2005). The accumulation of α-syn in brain regions involved in regulating sleep physiology is considered to be highly linked to disrupted sleep and circadian rhythm in PD patients (Rothman and Mattson, 2012; Kalaitzakis et al., 2013). A retrospective clinical pathological study revealed higher levels of α-syn burden in specific sleep-related brain regions in PD patients who also suffered from disrupted sleep compared with those without sleep disturbances (Kalaitzakis et al., 2013). The affected regions included: the locus coeruleus and the raphe nuclei, which are responsible for regulating REM, non-REM sleep, and the transition between them; the posterior hypothalamus, which is associated with somnolence; and the thalamus, which has been shown to be pivotal in organizing the sleep–wake rhythm (Reeves et al., 2006; Boeve et al., 2007). Other brainstem regions involved in regulating the sleep–wake cycle and arousal also showed lesions preceding the onset of motor symptoms in PD patients (Grinberg et al., 2010). Consistent with these clinical findings, accumulating evidence from animal studies lends further support to the notion that PD-related features are associated with changes to sleep parameters. For instance, one study found that mice overexpressing α-synuclein show increased α-syn burden and decreased firing rate in SCN neurons, which attenuates the ability of these neurons to transmit neural and hormonal signals from the SCN pacemaker (Kudo et al., 2011). Additionally, there were no defects in circadian parameters in transgenic MitoPark mice with slow and progressive degeneration of midbrain dopaminergic neurons prior to 19 weeks of age (Fifel and Cooper, 2014). However, after this age, DA levels significantly decreased in the midbrain, eventually declining to nearly 3% of their initial level, and all circadian parameters were markedly deteriorated. One explanation for these results given the known circadian rhythm of dopaminergic activity is that the dopaminergic signaling system transmits the circadian signal originating in the SCN to downstream target neuronal networks (Fifel and Cooper, 2014). Together, α-syn deposition and dopaminergic neuron loss are pivotal in PD-related pathological alteration and have been shown to be contributing factors to the disruption of sleep patterns and disturbances in circadian rhythm.

Melanopsin-containing retinal ganglion cells (mRGCs) are responsible for the non-image vision forming pathways projecting to different CNS regions and mediate several physiological responses, including the entrainment of circadian rhythms to the light–dark cycle, acute control of locomotor activity, sleep regulation, and the pupillary light reflex (Ksendzovsky et al., 2017). It is well documented that mRGCs innervate the SCN, which synchronizes circadian rhythms, as well as the lateral hypothalamus and the ventrolateral preoptic nucleus, which are jointly reponsible for regulating sleep behaviors (Münch and Kawasaki, 2013; La Morgia et al., 2017). Recently, a study analyzing the retinas of 24 donors demonstrated an age-associated reduction in the number of mRGCs and their extent of dendritic degeneration (Esquiva et al., 2017). Given that elderly individuals frequently suffer from sleep–wake disorders, it is reasonable to hypothesize that these changes to mRGC number and morphology may account for the incidence of circadian rhythm disorders in the aged (Esquiva et al., 2017). Post-mortem studies indicate that the retinal melanopsin-positive system degenerates in PD, as revealed by the significantly decreased density and morphological alterations of mRGCs in PD patients compared with controls (Ortuño-Lizarán et al., 2018). The reduction in the number of dopaminergic amacrine cells and the loss of synaptic connections with melanopsin cells in PD pathology may be responsible for the degeneration of mRGCs (Ortuño-Lizarán et al., 2020), given the bidirectional correlation between dopaminergic system and mRGCs. Previous studies have shown that mRGCs are modulated by DA released from dopaminergic cell innervations (Van Hook et al., 2012; Liao et al., 2016), while mRGCs are responsible for the continuous dopaminergic response to light (Prigge et al., 2016).

Recent studies using transgenic animals have offered promising insights into how the hypothetical factors contributing to PD-related pathophysiological processes drive circadian misalignment. The decreased expression levels of *Bmal1* and *Bmal2* have been reported in PD patients, indicating a potential correlation underlying PD-related processes and desynchronization of the circadian system (Cai et al., 2010; Ding et al., 2011). In support of this hypothesis, results from animal experiments also show that the PD-related genetic phenotype could impact sleep parameters. Mutations in *mul1* and *parkin* in Drosophila are sufficient to induce mitochondrial impairment and mitophagy, which are molecular symptoms observed in PD models, as well as being typical motor symptoms of PD (Kitada et al., 1998; Yun et al., 2014). Altered circadian rhythm and disrupted expression of clock genes were observed in a recent study conducted on mutants carrying mutations of both *mul1* and *parkin*, indicating a bidirectional correlation between PD-related pathology and circadian dysfunction (Ortuño-Lizarán et al., 2018). The level of ROS in the mutants was found to be higher than that in controls, consistent with decreased expression levels of the clock genes *clock* and *tim*. Notably, no significant difference was found in the expression rhythm of the *per* gene between mutants and controls, whereas the rhythm of the PER protein was completely disturbed (Doktór et al., 2019). Potential mechanisms underlying the difference between *per* transcription and PER translation could be ROS-induced alterations to the expression profiles of translational factors such as eIF2α, which enhance the expression of the PER protein (Lee et al., 2018). It is reasonable to propose that mutations in *mul1* and PARKIN which have a causal link with mitochondrial dysfunction and which have been shown to be involved in the development of PD impact circadian rhythms and the clock *via* multiple molecular mechanisms at both the transcriptional and translational level.

A study conducted on familial PD models indicated that dysfunction of the ER could also be a contributing factor to defects in sleep patterns and circadian rhythm (Lee et al., 2018). Further research has demonstrated that impaired neuropeptide distribution in specific neurons is responsible for disrupted circadian rhythm and sleep patterns in PD mutant *Drosophila* and has mapped these defects to specific neuronal clusters, including ventral lateral neurons and insulin-producing cells (Valadas et al., 2018). The LNvs are analogous to neurons in the human hypothalamus (Kunst et al., 2015), and secrete the vasoactive intestinal peptide (VIP)-like neuropeptide pigment dispersing factor (PDF), which is involved in regulating the circadian cycle (Muraro et al., 2013; Jones et al., 2018; Hamnett et al., 2019). Further investigations have revealed that the excessive ER–mitochondrial contact involved in phosphatidylserine transport interfere with the production of PDF-loaded dense core vesicles *via* effects on lipid membrane composition, resulting in a defect in the release of PDF and subsequent sleep pattern defects in flies (Lee et al., 2018). Notably, supplementation with PtdSer was shown to rescue sleep defects in PD models, indicating that the disturbed sleep and circadian rhythmicity can be attributed to neuronal dysfunction rather than neurodegeneration, and may therefore be reversible.

In conclusion, findings from both clinical and *in vivo* testing have lent strong support to the notion that PD-related processes are causally linked to poor sleep and disrupted circadian rhythms. In turn, disturbed sleep and circadian rhythms might act as possible early markers of neuropathology during the prodromal period of PD. However, further clinical studies investigating sleep parameters should be conducted on normal elderly individuals. These would help to ascertain whether PD-related pathological alterations, such as increased α-syn burden, are pivotal in the induction of sleep disturbance independent of the adverse effects of typical PD motor symptoms and of dopamine supplements taken by PD patients.

## Discussion

Clinical trials have demonstrated that patients with disturbed sleep patterns or circadian misalignment in old age more likely to exhibit pathological changes related to PD, along with an increased risk of PD. Evidence from further experimental studies has supported these findings with reports of adverse effects induced by sleep disruption, including increased levels of biomarkers related to PD, increased CNS oxidative stress, and inefficient removal of the PD-promoting metabolite α-syn. Additionally, patients with increased levels of α-syn burden in specific brain regions show disrupted sleep. These findings are in line with those from animal models overexpressing α-syn or demonstrating a progressive degeneration of DA neurons, which show gradual deterioration in sleep and circadian rhythm, indicating a direct causal link between PD-related processes and sleep disturbances. Taken together, these results suggest a bidirectional relationship between PD and sleep disruption, and as such, sleep has been widely considered to serve a disease-modifying role in the management of PD.

Further efforts should be made to disentangle the contribution of sleep disturbance and PD-related pathological alterations to their bidirectional correlation. For instance, longitudinal studies conducted on the general populationare warranted. These would preferably examine groups of patients with sleep complaints initiated preceding the clinical onset of PD across multiple clinical centers. Developments in non-invasive brain imaging methods and the successful identification of biomarkers may bring great benefits to advancing the assessment of sleep, circadian parameters, and the pathological progression of PD in human studies. Additionally, animal models incorporating broadly affected regions in the CNS are needed, given that current animal models selectively targeting the dopaminergic system fail to demonstrate the holistic sleep and circadian defects typical of the disease (Fifel et al., 2016).

Therapeutic managements targeting circadian rhythm have been widely proposed as being conducive to alleviating symptoms and disease burden in patients with PD. The utilization of bright light therapy has been shown to offer short-lived benefits with regard to consolidating activity rhythms or sleep patterns in patients with AD and PD (McCurry et al., 2011; Videnovic et al., 2017). The potential therapeutic effect of timed melatonin supplements has also been studied in PD patients, based on its antioxidant and apoptotic properties in *in vitro* and animal models. However, at present, no firm conclusion has been reached in terms of the therapeutic effects of light exposure or melatonin administration on motor symptoms, which can likely be attributed to methodological inconsistencies across trials. Therefore, it is important to carefully consider the methodological details of such studies and future longitudinal investigations evaluating the long-term therapeutic effects of circadian-oriented therapy on PD progression are warranted.

## Author Contributions

SC and AX planned the study. ZY, XZ, and CL analyzed the data and edited the manuscript. ZY wrote the manuscript. All authors contributed to the article and approved the submitted version.

## Conflict of Interest

The authors declare that the research was conducted in the absence of any commercial or financial relationships that could be construed as a potential conflict of interest.
